# Bioactive Phytocompound Profiling and the Evaluation of Antioxidant, Antihyperglycemic, and Antimicrobial Activities of Medicinal Plants from Serbian Traditional Medicine

**DOI:** 10.3390/molecules31010197

**Published:** 2026-01-05

**Authors:** Milica Aćimović, Anja Vučetić, Jelena Vulić, Aleksandra Ranitović, Teodora Marić, Vanja Travičić, Olja Šovljanski

**Affiliations:** 1 Institute of Field and Vegetable Crops, Novi Sad—National Institute of Republic of Serbia, Maksima Gorkog 30, 21000 Novi Sad, Serbia; 2 Faculty of Technology Novi Sad, University of Novi Sad, Bulevar Cara Lazara 1, 21000 Novi Sad, Serbia; anja.saveljic@uns.ac.rs (A.V.); jvulic@uns.ac.rs (J.V.); a.ranitovic@uns.ac.rs (A.R.); teodora.cvanic@uns.ac.rs (T.M.); vanjaseregelj@tf.uns.ac.rs (V.T.); oljasovljanski@uns.ac.rs (O.Š.)

**Keywords:** *Galium verum*, *Filipendula vulgaris*, *Lythrum salicaria*, *Sideritis montana*, *Teucrium chamaedrys*, *Teucrium montanum*

## Abstract

Medicinal plants represent an important source of bioactive compounds whose composition and biological activity are strongly influenced by geographical origin and extraction conditions. In this study, six medicinal plants traditionally used in south-eastern Serbia (*Galium verum*, *Filipendula vulgaris*, *Lythrum salicaria*, *Sideritis montana*, *Teucrium chamaedrys*, and *Teucrium montanum*) were investigated for their phytochemical composition and antioxidant, antihyperglycemic, and antimicrobial activities. Aqueous and 40% ethanol extracts were prepared and analyzed for total phenolic content (TPC) and total flavonoid content (TFC), followed by HPLC-DAD profiling of individual polyphenolic compounds. Antioxidant activity was assessed using DPPH, ABTS, and reducing power assays, antihyperglycemic activity by α-glucosidase inhibition, and antimicrobial activity by the microdilution method against selected bacterial and fungal strains. *L. salicaria* exhibited the highest TPC (113.56–119.09 mg GAE/g DW), while *F. vulgaris* showed the highest TFC (65.74–66.31 mg RE/g DW). HPLC analysis revealed notable levels of ferulic acid in *L. salicaria* ethanol extract (39.12 mg/g DW), as well as rutin, luteolin, and myricetin in several species. Ethanol extracts generally demonstrated stronger antioxidant activity, with *L. salicaria* showing the highest DPPH (378.60 µM TE/g) and reducing power (684.06 µM TE/g), while its aqueous extract exhibited the highest ABTS activity (3621.93 µM TE/g). Strong antihyperglycemic activity was observed for *F. vulgaris* extracts (100% α-glucosidase inhibition). Antimicrobial assays revealed higher sensitivity of Gram-positive bacteria, particularly *Listeria monocytogenes* and *Staphylococcus aureus*, with *F. vulgaris* and *L. salicaria* extracts showing the strongest effects. These findings highlight the significant influence of plant species and extraction solvent on bioactivity and support the potential of selected Serbian medicinal plants as sources of multifunctional natural bioactive compounds.

## 1. Introduction

Indigenous medicinal plants have long been recognized as valuable sources of bioactive compounds, many of which have inspired modern pharmaceuticals [1,2]. In recent decades, increasing incidence of chronic non-communicable diseases such as diabetes mellitus, oxidative stress–related disorders, and the rapid emergence of antimicrobial resistance have renewed scientific interest in plant-derived natural products as safer and multifunctional therapeutic alternatives [3,4]. Unlike synthetic drugs, phytochemicals often exert pleiotropic biological effects and may offer complementary or preventive benefits when used as functional foods or nutraceuticals [5,6].

Traditional medicine represents an important starting point for the identification of biologically active plants; however, ethnobotanical use alone does not provide sufficient evidence of efficacy, safety, or mechanisms of action [7,8]. Many medicinal plants used in Serbian traditional medicine, particularly those from rural and mountainous regions, remain insufficiently investigated using modern phytochemical and bioactivity-oriented approaches [9,10,11,12,13,14,15,16,17]. Existing studies often focus on isolated compounds or single biological activities, leaving a significant gap in comprehensive evaluations that integrate phytochemical profiling with multiple relevant biological assays.

Eastern Serbia, particularly the Sokobanja region, preserves a rich ethnopharmacological heritage in which bitter medicinal plants play a prominent role [18,19]. Bitterness in plants is frequently associated with the presence of phenolic compounds, flavonoids, iridoids, and terpenoids—classes of secondary metabolites known for antioxidant, antihyperglycemic, and antimicrobial activities. Despite their long-standing use, many of these species lack systematic studies that correlate their chemical composition with experimentally validated biological effects.

In this context, six traditionally used medicinal plants: lady’s bedstraw (*Galium verum*), dropwort (*Filipendula vulgaris*), purple loosestrife (*Lythrum salicaria*), mountain ironwort (*Sideritis montana*), wall germander (*Teucrium chamaedrys*), and mountain germander (*Teucrium montanum*) were selected for investigation. Although individual aspects of these plants have been sporadically reported in the literature, a comparative and integrative analysis of their phytochemical profiles and key biological activities remains limited or absent. This lack of comprehensive data restricts their potential translation into evidence-based applications in modern phytotherapy.

Two types of extracts were prepared in order to assess how extraction polarity influences the recovery of bioactive compounds and the resulting biological activities. Aqueous extracts reflect traditional modes of preparation, while organic solvent extracts are commonly employed to enhance the extraction of phenolic and other semi-polar constituents, thereby enabling a more complete evaluation of the plants’ bioactive potential.

The aim of this study was therefore to comprehensively characterize the phytochemical composition of selected medicinal plants from Serbian traditional medicine and to evaluate their antioxidant, antihyperglycemic, and antimicrobial activities. We hypothesized that (i) these plants contain diverse phenolic and related bioactive compounds, (ii) their biological activities differ depending on extraction solvent, and (iii) specific phytochemical profiles are associated with enhanced biological effects. By integrating ethnopharmacological knowledge with modern analytical and biological approaches, this study seeks to contribute to the scientific validation of traditional medicinal plants and to identify candidates for further development as functional ingredients or therapeutic leads.

## 2. Results

This study provides a comparison of the phytochemical composition and biological activities of six medicinal plants traditionally used in south-eastern Serbia. By analyzing both aqueous and ethanol extracts, we identified significant differences in total phenolic and flavonoid contents, individual polyphenolic profiles, antioxidant capacities, antihyperglycemic activities, and antimicrobial effects.

### 2.1. Bioactive Compounds from Aqueous and Ethanol Extracts

Table 1 shows the content of TPC and TFC in water and ethanol extracts of selected plants. *L. salicaria* had the highest content of TPC (113.56–119.09 mg GAE/g, depending on the extract type), followed by *F. vulgaris*, *T. chamaedrys*, *G. verum*, and *T. montanum*, while the lowest TPC was recorded in *S. montana* (10.01–12.66 mg GAE/g). For *F. vulgaris*, *G. verum* and *T. chamaedrys*, TPC was higher in water than in ethanol extract, whereas in the case of *L. salicaria*, *S. montana*, and *T. montanum*, the ethanolic extract was richer in TPC. *F. vulgaris* had the highest content of TFC (65.74–66.31 mg RE/g DW, depending on the extract type), followed by *T. chamaedrys*, *G. verum*, *L. salicaria*, and *T. montanum*, while the lowest TFC, as in the case of TPC, was noted in *S. montana* (4.53–5.12 mg RE/g).

### 2.2. HPLC Analysis

The aqueous and ethanol extracts were examined for 26 phenolic compounds using the HPLC method, identifying 12 compounds and quantifying their levels (Table 2). A total of five phenolic components were identified in both *F. vulgaris* extracts (caffeic and gallic acid, rutin, myricetin, and luteolin), while gentisic acid was confirmed only in the aqueous extract. Flavonoids (rutin, myricetin, and luteolin) were confirmed in both extracts of *G. verum*, while caffeic acid and *p*-hydroxybenzoic acid were present only in the aqueous extract, and ferulic acid was present only in the ethanol extract. Only one phenolic compound, ferulic acid, was identified in the ethanolic extract of *L. salicaria*, and it was present in a high concentration (39.12 mg/g DW), while the presence of two phenolic acids, gallic and protocatechuic, was found in the aqueous extract, with amounts of 7.95 mg/g and 6.40 mg/g DW, respectively. A significantly different phenolic profile was also recorded in *S. montana*, where flavonoids were present in the aqueous extract, and only myricetin from this group of compounds, along with caffeic and syringic acids, were detected in the ethanol extract. The same is true for *T. chamaedrys*, where caffeic, gentisic, and ferulic acids were recorded in the aqueous extract, and gallic and syringic acids, as well as myricetin, in the ethanol extract. The presence of sinapic acid was found in both *T. montanum* extracts, while gallic acid was also present in the water extract, and ferulic and rosmarinic acids in the ethanol extract.

### 2.3. Antioxidant Activity of Aqueous and Ethanol Extracts

Employing multiple antioxidant assays with distinct mechanisms of action provides a comprehensive understanding of plants’ antioxidant potential, as each assay targets different aspects of antioxidant behavior. Accordingly, the antioxidant activity of the prepared aqueous and ethanol extracts was evaluated using three complementary assays DPPH and ABTS radical scavenging activities, and the reducing power assay (Table 3). The results revealed that ethanol extracts generally exhibited higher antioxidant activities in the DPPH and reducing power assays, with one exception: the *G. verum* extracts, where DPPH results were comparable between aqueous and ethanol extracts. However, the reducing power assay differentiated these extracts by approximately 10 µM TE/g. Conversely, the ABTS assay demonstrated higher activity in aqueous extracts, except for *S. montana*, where the ethanol extract showed superior performance. This variability is likely attributable to the presence of specific phenolic compounds and other antioxidants in each extract, as well as their distinct mechanisms of action against free radicals. Among the tested extracts, *L. salicaria* demonstrated exceptional antioxidant potential. Its ethanol extract achieved the highest DPPH and reducing power values, measured at 378.60 µM TE/g and 684.06 µM TE/g, respectively. Additionally, its aqueous extract exhibited the highest ABTS activity among all tested extracts, at 3621.93 µM TE/g. In contrast, the *S. montana* aqueous extract displayed the lowest activities across all assays, with DPPH, reducing power, and ABTS activities measured at 26.45 µM TE/g, 16.80 µM TE/g, and 16.80 mM TE/g, respectively.

### 2.4. Antihyperglycemic Activity of Aqueous and Ethanol Extracts

The antihyperglycemic activities of the selected plants were determined and expressed as percentages at adequately diluted concentrations for each plant as observed in Table 4. The preparation of dilutions was tailored based on the strength of the extracts’ inhibition of α-glucosidase activity to ensure accurate and clear results. Stronger extracts were diluted more to standardize measurements and avoid saturation effects. The most pronounced antihyperglycemic activity was observed in both ethanol and aqueous extracts of *F. vulgaris*, reaching 100% even at particularly low concentrations. Conversely, the aqueous and ethanol extracts of *G. verum* exhibited the lowest activity at 10.68% and 37.17% at a significantly higher concentration (3.77 mg/mL). Generally, ethanol extracts displayed stronger antihyperglycemic activities than aqueous extracts, with one notable exception, the aqueous extract of *T. chamaedrys*, which exhibited higher activity than its ethanol counterpart. This could correlate with activities of phenolic acids that were present only in aqueous extract. Among the ethanol extracts, moderate antihyperglycemic activity was observed in the *S. montana* extract (37.70% inhibition of α-glucosidase at 1.87 mg/mL), followed by the *T. montanum* and *T. chamaedrys* extracts, with inhibition rates of 11.04% and 9.77%, respectively. Notably, at the same concentration, both extracts of *L. salicaria* stood out for their relatively high antihyperglycemic activity, further highlighting its potential.

### 2.5. Antimicrobial Activity of Aqueous and Ethanol Extracts

Table 5 provides a comprehensive summary of the minimal bactericidal concentration (MBC) of aqueous and ethanol extracts of six plant species, as well as their minimal fungicidal concentration (MFC) against yeasts and fungi. The inability to determine the MFC within the tested concentration range suggests a pronounced resistance of eukaryotic organisms to these plant extracts.

The MBC values varied from 3.125 to 50 mg/mL, reflecting differing susceptibilities among the tested bacterial strains. Notably, Gram-positive bacteria demonstrated greater sensitivity to the extracts compared to Gram-negative bacteria. Among the evaluated plants, *F. vulgaris* and *L. salicaria* exhibited the strongest antimicrobial effects. The most sensitive bacterial strains were *L. monocytogenes* and *S. aureus*. For example, the MBC values for *L. monocytogenes* were 3.125 mg/mL for the ethanol extract of *L. salicaria* and 6.25 mg/mL for its aqueous extract, as well as the aqueous extract of *F. vulgaris*. Similarly, *S. aureus* showed an MBC of 3.125 mg/mL for the aqueous extract of *L. salicaria*. The pronounced sensitivity of *L. monocytogenes* is particularly noteworthy due to its relevance as a critical foodborne pathogen. In contrast, *E. coli* displayed the highest resistance, with MBC values consistently ≥50 mg/mL across all tested extracts. Other bacterial strains exhibited intermediate levels of resistance, with MBC values ranging from 25 to 50 mg/mL. Aqueous and ethanol extracts of the same plant showed comparable activity against certain bacterial strains. For instance, both extracts of *F. vulgaris* demonstrated similar efficacy against *E. coli*, *P. aeruginosa*, and *E. faecalis*. Similarly, extracts of *L. salicaria* showed equal activity against *E. coli*, *S.* Typhimurium, and *E. faecalis*. However, significant variations in antimicrobial potency between aqueous and ethanol extracts were observed. For example, the MBC of the aqueous extract of *F. vulgaris* against *S.* Typhimurium was 50 mg/mL, while the ethanol extract reduced this value to 25 mg/mL. A more pronounced difference was evident in the case of *S. aureus*, where the MBC of the aqueous extract of *L. salicaria* was 3.125 mg/mL, compared to 25 mg/mL for its ethanol extract. *G. verum* extracts exhibited minimal antibacterial potential, with MBC values exceeding 50 mg/mL against most bacterial strains, except for the ethanol extract against *E. faecalis*, which showed an MBC of 50 mg/mL. Similarly, *T. chamaedrys* displayed low antimicrobial activity, with an MBC of 50 mg/mL against *E. coli*, *S.* Typhimurium, and *S. aureus*, but failed to show activity within the tested concentration range for other strains. According to Table 5, the obtained results indicate the variability in plant-derived antimicrobial efficacy and the notable resistance of Gram-negative bacteria and eukaryotic organisms. The data underscore the potential of certain plant extracts, such as those of *F. vulgaris* and *L. salicaria*, as promising sources of antibacterial agents, particularly against Gram-positive foodborne pathogens like *L. monocytogenes* and *S. aureus*.

## 3. Discussion

### 3.1. Bioactive Compounds from Different Extracts

Selected plants may be considered promising sources of bioactive compounds for pharmaceuticals, nutraceuticals, functional foods, and dietary supplements. Phytochemical analyses show that all selected species contain significant amounts of polyphenolics [20,21,22,23,24,25,26,27,28,29,30,31]. In practice, the concentrations of TPC and TFC depend strongly on the type of solvent and the extraction technique used [32]. A brief overview of TPC and TFC values for the selected plants is presented in Table 6. As shown, *G. verum* exhibits particularly large variability, ranging from very low to exceptionally high phenolic levels depending on the method and solvent. Other plants, such as *F. vulgaris*, *L. salicaria*, *S. montana*, *T. chamaedrys*, and *T. montanum*, also show substantial phenolic and flavonoid contents, especially in methanolic or ethanol-based extracts. Overall, the data confirm these species as rich sources of phenolic and flavonoid compounds, with extraction conditions playing a key role in determining yield.

In comparison, variations in TPC and TFC between aqueous and ethanol extracts in this study are less distinct, most likely due to the closer similarity of polarity of the used solvents. The most notable differences are observed within extracts of *T. chamaedrys* (TPC and TFC) and *T. montanum* (TFC). Since both solvents were chosen to reflect the traditional way of preparation and usage of these plants in Serbia, the similar and abundant content of TPC and TFC is considered advantageous. Consistent with these findings, it has been reported that aqueous and ethanolic extracts are often comparable in terms of TPC, particularly in plants traditionally consumed as infusions [33], despite the general observation that antioxidant extraction efficiency is strongly influenced by solvent composition. The highest concentration of TPC, both found and confirmed in the literature report, belonged to *L. salicaria*. Meanwhile, *F. vulgaris* had the highest amount of TFC, closely followed by *T. chamaedrys*. As it was presented in Table 1, all examined plants showed valuable contents of total phenolics and flavonoids comparing to literature data. In our study, *G. verum* had slightly higher TPC using aqueous solvent, while TFC showed opposite results while comparing aqueous and ethanol solvents. *F. vulgaris* expressed almost the same results for TPC and TFC for both solvents. TPC with ethanol solvent were slightly higher, while opposite results were obtained for TFC with aqueous solvent of *L. salicaria*. TFC with ethanol were higher in *S. montana* and *T. montanum*, while TPC were reverse. *T. chamaedris* expressed higher contents for aqueous solvent, while ethanol was better for TFC. The obtained findings indicate that plant species have a greater impact on phenolic and flavonoid content than solvent choice, which aligns with previous phytochemical research.

### 3.2. HPLC Profiles

A large number of phenolic compounds are found in selected medicinal plants. In the methanol extract of *F. vulgaris*, a total of 12 organic acids and phenolic compounds were identified, with gallic acid having the highest content (925.67 µg/g extract) [34]. Moreover, the main phenolic compounds identified in the aerial parts of *F. vulgaris* are flavonoid aglycones and glycosides, phenolic acids, and hydrolyzable tannins [35]. In the *G. verum* herb, hydroxycinnamic derivatives, flavonoids, and polyphenols are responsible for its biological activity [36]. The polyphenolic profile of the *L. salicaria* extract is extremely complex, with vitexin and orientin, as well as their isomers, several phenolic acids, and tannins, with significantly higher content in the flowering tops than in the leaves or shoots [37,38]. Among the polyphenols in *S. montana*, flavonoids were found to be the major bioactive compounds, especially morin, catechin, and naringenin [39,40]. Analysis showed that *Teucrium* species are rich in phenolic compounds, mainly hydroxycinnamic acid derivatives, phenylethanoid glycosides, flavonoid glycosides, and flavonoid aglycones [41]. Table 7 provides a review of polyphenolic profiles of different extracts from *F. vulgaris*, *G. verum*, *L. salicaria*, *S. montana*, *T. chamaedrys*, and *T. montanum*.

As can be noted, analysis of polyphenolic compounds was extensively analyzed in the previous studies. Still, depending on the geographical region, the content of bioactive compounds can vary quantitatively as well as qualitatively. Therefore, observed quantitative differences are expected, with overall phenolic profiles and dominant compound classes still in agreement. In general, the HPLC results indicated noticeable differences between aqueous and ethanolic extracts that likely arose from solvent-steered selectivity towards specific phenolic subclasses. And while water favored the extraction of more polar phenolic acids, recovery of less polar hydroxycinnamic acids and flavonoids, including aglycone-associated forms, was more prevalent in ethanol solution. For instance, ethanolic extracts of *L. salicaria* and *S. montana* contained higher levels of ferulic and syringic acids, respectively, whereas aqueous extracts of *G. verum* and *F. vulgaris* showed higher contributions of caffeic and gallic acids, along with distinguishable differences in flavonoid levels among the investigated plant species. Namely, myricetin and luteolin were quantified in higher amounts in ethanol solutions od *G. verum*, *F. vulgaris* and *S. montana* plants (Table 2). This was also demonstrated in previous studies, where the phenolic composition was strongly influenced by solvent polarity, and ethanol/water systems selectively extracted different classes of phenolics and flavonoids dependent on their chemical structure and solubility [47,48,49]. Both extracts across all plant species presented a favorable polyphenol composition, supporting the relevance of traditional herbal preparation practices.

### 3.3. Antioxidant Activity

A brief review of the antioxidant potential of the six investigated plants is shown in Table 8. The reviewed species exhibit notable antioxidant activity across various assays (DPPH, ABTS, FRAP, and reducing power), with *G. verum* showing particularly strong radical-scavenging effects depending on the extraction solvent. *F. vulgaris*, *L. salicaria*, *S. montana*, *T. chamaedrys*, and *T. montanum* also demonstrate substantial antioxidant potential, especially in methanolic and ethanolic extracts. Overall, the results highlight solvent-dependent variability but consistently confirm these species as rich sources of natural antioxidants.

In this study, a comprehensive antioxidant profiles of aqueous and ethanol extracts were obtained using three antioxidant assays (DPPH, ABTS, and RP). *L. salicaria* expressed the highest antioxidant activities, followed by *F. vulgaris* and *T. chamaedrys*. These findings are consistent with the literature findings and directly corroborate the previously determined results of total phenolics and flavonoid content, which greatly contribute to antioxidant activity. The strongest antioxidant activity of *L. salicaria* extracts can be attributed to high contents of ferulic and gallic acids and myricetin, compounds widely recognized for their strong radical scavenging potential [50]. In general, the results seem to indicate that the variations in antioxidant activity between aqueous and ethanol extracts do not follow a clear trend and are more dependent on plant species than on the solvent used. This is most likely connected to unique polyphenolic profile of each plant and their polarity-driven extraction of compounds in each solvent.

### 3.4. Antihyperglycemic Activity

The literature provides substantial data on the antihyperglycemic activity of plants belonging to the genera *Galium*, *Filipendula*, *Lythrum*, *Sideritis*, and *Teucrium*. However, when considering locally selected plants, the available literature becomes more limited. Despite this, there are noteworthy examples that support the potential of these plants in managing diabetes. For instance, *G. tricornutum* has been shown to possess significant antidiabetic potential [51]. Similarly, a flavonoid glycoside isolated from the ethanol extract of *G. verum* exhibited potent intestinal α-glucosidase inhibitory activity in vitro, with an IC_50_ of 13.7 μM [52]. These findings highlight the importance of *Galium* species in diabetes management.

Herbal teas from other plants in the *Filipendula* genus, such as *F. ulmaria*, *F. camtschatica*, *F. denudata*, and *F. stepposa*, have also demonstrated significant potential in inhibiting amylase, α-glucosidase, and advanced glycation end product formation [53]. Since these species belong to the same genus, it could be inferred that their properties align with our findings and support the antidiabetic potential of *Filipendula* species. In addition, ethyl ether extract of *L. salicaria* has shown significant hypoglycemic activity in animals with glucose- and epinephrine-induced hyperglycemia, as well as in alloxan- and streptozotocin-diabetic rats and mice [54]. This demonstrates the promising therapeutic applications of *Lythrum* species in the treatment of diabetes.

Extracts from *Sideritis* species have also been explored for their antihyperglycemic properties. Petroleum ether and ethanol extracts of *S. taurica* have exhibited significant antihyperglycemic activity [55], and *S. pisidica* hexane extract has similarly shown promise [56]. Furthermore, *Teucrium* species, such as *T. polium*, *T. leucocladum*, and *T. alyssifolium*, have all demonstrated antidiabetic effects. *T. polium* aqueous extracts have been shown to possess antidiabetic properties [57], while *T. leucocladum* exhibited antihyperglycemic effects in streptozotocin-induced diabetic rats [58]. Additionally, *T. alyssifolium* exerts its antidiabetic effect by enhancing glucose uptake and suppressing glucose absorption [59]. Additionally, investigations from Balkan region have shown the antihyperglycemic activity of different extracts of *T. montanum* and *T. chamaedrys* from Bosnia and Herzegovina (Ozren Mt.) and the eastern part of Serbia (Vlasina and Vrmdža) [31,60].

Collectively, these studies provide compelling evidence of the therapeutic potential of these locally selected plants in managing diabetes, supporting their continued investigation and use in traditional and modern medicine. A brief review of the antihyperglycemic activity of the six investigated plants is presented in Table 9.

In our study, as expressed in Table 4, ethanol extracts of *G. verum*, *L. salicaria*, *S. montana* and *T. montanum*, expressed better activities in made tests, while only *T. chamaedrys* showed better results in aqueous solutions. As presented in Table 9, *T. montanum* showed better results using ethanol than water for extraction. Also, *G. verum* showed positive activity using ethanol (Table 9). The significance of plant-derived antihyperglicemic compounds lies in the discovery of natural bioactive compounds that could provide accessible and cost-effective therapeutic options for diabetes prevention and management.

### 3.5. Antimicrobial Activity

The aqueous and ethanol extracts of the selected plants showed variable antimicrobial activity, with antibacterial effects generally stronger than antifungal effects (Table 5). This variability is influenced by plant species, extraction methods, solvent, and the target microorganism [61,62]. The antimicrobial activity of the plant extracts is linked to bioactive secondary metabolites, particularly phenolics and flavonoids, which were most abundant in *F. vulgaris* and *L. salicaria*, correlating with their strong antimicrobial potential. Higher sensitivity of Gram-positive bacteria compared with Gram-negative bacteria was also reported by other authors [29,63]. The higher resistance of Gram-negative bacteria could be explained by the complex structure of the bacterial wall, which makes it impervious to active compounds [43].

In this study, *G. verum* showed low antimicrobial potential, in contrast to a previous report indicating some activity; however, even in that study, the water extract demonstrated limited biological effectiveness [43]. Currently, there is a lack of research on the antimicrobial properties of *F. vulgaris* extracts, with existing studies limited to its essential oil [64]. The results of the antimicrobial activity of the methanol extract of *L. salicaria* tested by other authors show more pronounced antimicrobial activity, with MIC values several times lower than those found in this study [29,65]. In contrast to our findings, where *S. montana* exhibited no activity within the tested concentration range, previous studies have reported antimicrobial potential for this plant [63,66].

In this study, *T. chamaedrys* extracts exhibited low antimicrobial potential, which aligns with findings indicating weak activity (MIC ≥ 150 mg/mL) [62]. In contrast, another study reported stronger antimicrobial effects, with MIC values ranging from 0.15 to >20 mg/mL depending on the strain and extract type [61]. Unlike the results obtained in this study, where *T. montanum* did not show activity in the examined concentration range, other authors showed the antimicrobial potential of these plants [61,67]. Variability in antimicrobial activity is observed both between studies and among different extracts of the same plant. This non-uniformity reflects differences in chemical composition, influenced by geographical origin, extract type, and the microbial strains tested. A brief overview of the antimicrobial activity of the plants studied is presented in Table 10.

As can be seen in Table 10, comparison of the obtained antimicrobial findings with literature reveals both consistencies and notable discrepancies. For instance, the weak efficacy observed for *G. verum* in this study is in line with earlier reports of only limited activity for its aqueous extracts. Similarly, *T. chamaedrys* exhibited negligible antimicrobial potency here, which agrees with one study reporting high MIC values for this species. However, another study found *T. chamaedrys* to be much more efficacious under different extraction conditions, highlighting an important discrepancy. In contrast to our results, *L. salicaria* showed substantially stronger activity in prior investigations using methanolic extracts, with MICs several-fold lower than those we observed. Likewise, *S. montana* and *T. montanum* exhibited no detectable antimicrobial effect within our tested concentration range, whereas previous studies have reported clear inhibitory activity for extracts of these plants. For *F. vulgaris*, direct comparison is limited due to scarce literature on its extracts (existing data focus mainly on the essential oil), so our findings provide novel insights but lack a published benchmark. These inter-study variations can be explained by differences in solvent polarity, plant chemotype, and microbial strain selection. The choice of extraction solvent critically determines the spectrum and yield of active phytochemicals; thus, studies employing more polar or non-polar solvents (e.g., methanol, acetone, or distinct fractions) may isolate different antimicrobial compounds than our aqueous or hydroethanolic extracts, leading to divergent MIC/MBC outcomes. The particular microbial strains and assay conditions used by each study further influence the results, since pathogen susceptibility can vary widely.

### 3.6. Perspectives of Traditional Medicinal Plants, Study Limitations and Implications

Traditional medicinal plants continue to attract considerable attention in modern pharmacology and nutrition due to their rich content of bioactive compounds [4,68]. Advances in extraction and analytical techniques now allow the identification and quantification of these compounds, enabling a better understanding of their mechanisms of action, including antioxidant, antimicrobial, anti-inflammatory, and antidiabetic effects [69,70]. In pharmacology, such insights are paving the way for the development of plant-based therapeutics, either as standardized extracts or as sources of lead compounds for drug design [71]. In the field of nutrition, these plants are increasingly recognized for their potential as functional foods or nutraceuticals, contributing to disease prevention and overall health maintenance [72]. Integrating traditional knowledge with modern scientific approaches offers promising avenues for sustainable healthcare solutions, personalized nutrition, and the discovery of novel bioactive compounds [73,74].

The findings of this study underscore the importance of evaluating plant species from specific geographical regions, as local climate and environmental conditions can influence the accumulation of bioactive compounds. While medicinal plants have been widely studied, the comparative analysis of these six Serbian species using multiple biological assays and detailed HPLC profiling provides novel insights into their relative antioxidant, antihyperglycemic, and antimicrobial potentials. This integrative approach allows for the identification of the most promising species and extracts for further research, offering a clearer purpose for the work beyond general phytochemical characterization.

However, this study has certain limitations. All analyses were conducted in vitro, and the bioactivities observed may not directly translate to in vivo or clinical outcomes. Only two extraction solvents were tested, and other solvents or extraction techniques could yield different results. Additionally, while the identified polyphenolic compounds and bioactivities indicate potential functional benefits, any claims regarding pharmaceutical applications are preliminary. Further studies, including in vivo experiments, safety assessments, and formulation studies, are required before any practical therapeutic applications can be considered. Despite these limitations, the current findings provide a solid foundation for future research and highlight species-specific differences in bioactive potential that could inform the development of functional foods or nutraceuticals.

## 4. Materials and Methods

### 4.1. Chemicals and Reagents

Analytical-grade reagents used in the experiments: 96% ethanol (Reachem, Srbobran, Serbia), Trolox, Folin–Ciocalteu reagent (2 M), gallic acid, rutin, trichloroacetic acid 2,2-diphenyl-1-picrylhydrazyl (DPPH), 2,2-azino-bis-3-ethyl benzathine-zoline-6-sulphonic acid (ABTS) from Sigma Chemical Company (St. Louis, MO, USA), pure methanol, sodium carbonate and potassium ferricyanide from Lachner (Neratovice, Czech Republic), ferric chloride (JT Baker, Deventer, The Netherlands), α-glucosidase enzyme from *Saccharomyces cerevisiae* (Sigma Aldrich, Buchs, Switzerland).

### 4.2. Plant Material Collection and Extraction

Six plants widely used in Serbian traditional medicine were collected in the Sokobanja region (Rtanj Mountain, Jošanica, and Milušinac villages) during July 2021. Voucher specimens were confirmed and deposited at the Herbarium of the Department of Biology and Ecology, Faculty of Sciences, University of Novi Sad (BUNS Herbarium), as follows: *Filipendula vulgaris* Moench [syn. *F. hexapetala* Gilib.] (2-1091), *Galium verum* L. (2-1097), *Lythrum salicaria* L. (2-1090), *Sideritis montana* L. (2-1092), *Teucrium chamaedrys* L. (2-1094), and *Teucrium montanum* L. (2-1407).

The above-ground parts of the selected plants were collected during the flowering phase, naturally dried in the shade until they reached a constant weight, and then placed in multilayer paper bags until further analysis. Prior to the experiments, the plant material was ground in an electric mill and used to prepare the extracts.

Selected medicinal plants were used for ultrasound extraction with two different solvents: distilled water (70 °C) and 40% ethanol. The extractions were performed using 1 g of plant material and 10 mL of solvents, with an ultrasound bath for 30 min (Elmasonic S15 H GmbH, Singen, Germany). Constant and default frequency of 50/60 Hz was used, while maintaining temperature under 40 °C. The supernatant was then centrifuged for 5 min, separated using a vacuum filtration bottle, and kept refrigerated until further analysis.

### 4.3. Identification of Bioactive Compounds from Extracts

Extracts from the investigated plants were subjected to spectrophotometric assays to determine their total phenolic (TPC) and flavonoid (TFC) contents. The microscale-adapted Folin-Ciocalteau method was used for TPC determination [75], while TFC was determined using the Markham method [76]. In brief, for TPC, absorbances were measured at a wavelength of 740 nm, alongside their blanks, and the results were calculated as gallic acid equivalents (GAE) per g of dried plant material. For TFC, absorbances were measured at a wavelength of 430 nm, alongside their blanks, and the results were calculated as mg rutin equivalent (RE) per g of dried plant material.

### 4.4. High-Performance Liquid Chromatography (HPLC) Analysis

Aqueous and ethanol extracts of *G. verum*, *F. vulgaris*, *L. salicaria*, *S. montana*, *T. chamaedrys*, and *T. montanum* were subjected to HPLC analysis coupled with DAD detector (Shimadzu Prominence, Kyoto, Japan), to determine and measure the polyphenolic constituents. Before analysis, prepared extracts were evaporated using a rotating vacuum pump (Rotavapor 2-210. Buchi, Flawil, Switzerland), dissolved in the mobile phase, and filtered through a 0.45 μm membrane. All of the analyses were run in triplicate and data acquisition was carried out by the LC Solution Software version 1.25 (Shimadzu, Kyoto, Japan). The A (acetonitrile) and B (1% formic acid) were used as mobile phases, with a flow rate of 1 mL/min and a gradient profile going from 10% to 25% A from 0 to 10 min; a linear increase to 60% A from 10 to 20 min; and a further linear increase to 70% A from 20 to 30 min, followed by a return to the initial 10% A over 10 min, with an additional 5 min for equilibration. For detection of present polyphenols, the separation was carried out on a Luna C-18 RP column (5 μm, 250 × 4.6 mm) from Phenomenex (Torrance, CA, USA), with a C18 guard column (4 × 30 mm) from the same supplier. Different wavelengths for specific compounds were used to record chromatograms (280 nm for hydroxybenzoic acids, 320 nm for hydroxycinnamic acids and 360 nm for flavonoids) and all results were calculated as mg per g of dried plant weight (DW). The identified results were validated using reference standards, which were dissolved in 50% methanol, filtered, and prepared at a concentration of 1 mg/mL. These standards, including gallic acid, protocatechuic acid, catechin, epicatechin, caffeic acid, ferulic acid, vanillic acid, syringic acid, coumaric acid, gentisic acid, rosmarinic acid, sinapic acid, chlorogenic acid, *p*-hydroxybenzoic acid, cinnamic acid, ellagic acid, salicylic acid, quercetin, kaempferol, rutin, myricetin, naringin, apigenin, luteolin, syringaldehyde, and sinapaldehyde, were investigated to ensure the reliability of the validation process.

### 4.5. Determination of Antioxidant Activity

In order to investigate the antioxidant potential of selected plants, the prepared extracts were subjected to three different antioxidant assays: 2,2-diphenyl-1-picrylhydrazyl (DPPH), 2,2′-azinobis-3-ethylbenzothiazoline-6-sulphonic acid (ABTS), and reducing power (RP). All three antioxidant tests were performed using spectrophotometry at different wavelengths and their antioxidant capacity was expressed as µmol of Trolox equivalent (TE) per g of dried plant material [77]. Briefly, for the DPPH assay, DPPH solution in methanol (0.89 mM, 250 μL) was added to a microplate well, along with the diluted extracts (10 μL), and measured after 50 min at 515 nm. Meanwhile, ABTS scavenging activity was measured by adding 250 μL of activated ABTS solution (with MnO_2_), measuring initial absorbances at 414 nm, and then adding 2 μL of extract, incubating at 25 °C for 35 min before measuring final absorbances. First step of reduction power (RP) activity included creating a reaction mixture of 75 μL of extract, sodium phosphate buffer pH 6.6, and 1% m/V potassium ferricyanide, before incubating it at 50 °C for 20 min. After the incubation and cooling, the same amount of 10% trichloroacetic acid was added to the mixture. The aliquot of 50 μL was added to the microtiter plate, along with 50 μL of distilled water and 10 μL of 0.1% ferric chloride solution, and the absorbances were measured at 700 nm.

### 4.6. Determination of Antihyperglycemic Activity

For the antihyperglycemic assay, after extraction and separation, the supernatant was evaporated using a rotating vacuum evaporator and then diluted in the buffer used for the test, to different concentrations depending on the extract. The antihyperglycemic activity of the newly prepared samples was observed using the enzyme α-glucosidase, 4-nitrophenyl α-D-glucopyranoside substrate, and 10 mM potassium phosphate buffer. The results were measured spectrophotometrically at 405 nm and expressed as percentages at specific concentrations [78].

### 4.7. Determination of Antimicrobial Activity

Antimicrobial activity was tested using the microdilution method [79,80], with the initial concentration of the extracts being 50 mg/mL. The bacterial strains used in this study were Gram-negative: *Escherichia coli* (ATCC 25922), *Salmonella enterica* subsp. *enterica* serovar Typhimurium (ATCC 14028), *Pseudomonas aeruginosa* (ATCC 27853), and Gram-positive: *Staphylococcus aureus* (ATCC 25923), *Listeria monocytogenes* (ATCC 35152), *Enterococcus faecalis* (ATCC 29212). The fungal strains included yeasts: *Saccharomyces cerevisiae* (ATCC 9763) and *Candida albicans* (ATCC 10231), and mold *Aspergillus brasiliensis* (ATCC 16404). All microbial strains were maintained at −80 °C in a deep freezer as part of the institutional Collection of Microorganisms. Prior to each experiment, cultures were reactivated by streaking onto appropriate solid media. Bacterial strains were subcultured on Mueller–Hinton agar (HiMedia Laboratories, Mumbai, India), whereas yeasts and filamentous fungi were subcultured on Sabouraud dextrose agar (HiMedia Laboratories, Mumbai, India). Bacterial cultures were incubated at 37 °C for 24 h, while yeasts and fungi were incubated at 25 °C for 72 h. For antimicrobial assays, sterile distilled water was used as the negative control, while amoxicillin or actidione was employed as the positive control.

### 4.8. Statistical Analysis

All tests were performed in triplicate, and the results are expressed as the mean value ± standard deviation (SD).

## 5. Conclusions

The present study demonstrates that the six investigated plant species possess distinct phytochemical profiles and biological activities, strongly influenced by both plant species and the type of extraction solvent. *L. salicaria* and *F. vulgaris* showed the highest levels of phenolic compounds and flavonoids, which corresponded to their superior antioxidant, antihyperglycemic, and antimicrobial properties. In contrast, *S. montana* consistently exhibited the lowest phenolic content and the weakest biological activity in all assays. The HPLC analysis revealed substantial qualitative and quantitative differences in phenolic composition between aqueous and ethanol extracts, which highlights the importance of solvent selection for optimizing the recovery of bioactive constituents.

The biological assays confirmed that ethanol extracts generally produced higher antioxidant and antihyperglycemic activities, whereas aqueous extracts frequently showed stronger ABTS radical scavenging potential. These results reflect the diverse mechanisms of action of phenolic compounds and other antioxidant molecules. *L. salicaria* exhibited particularly strong antioxidant activity across all methods, while *F. vulgaris* demonstrated the most pronounced inhibition of α-glucosidase, reaching complete inhibition even at low concentrations. These findings suggest that specific phenolic acids and flavonoids extracted with ethanol may play important roles in mediating antihyperglycemic and radical scavenging effects.

The antimicrobial evaluation indicated considerable variability among species and bacterial strains, with Gram-positive bacteria showing greater susceptibility than Gram-negative bacteria. Among the examined plants, *F. vulgaris* and *L. salicaria* displayed the strongest antibacterial effects, especially against foodborne pathogens such as *L. monocytogenes* and *S. aureus*. The lack of measurable activity against yeasts and fungi emphasizes the high resistance of these organisms to the tested extracts. Overall, the results highlight the potential of selected plant species, particularly those rich in phenolic compounds, as promising natural sources of antioxidant, antihyperglycemic, and antibacterial agents suitable for further development in food, pharmaceutical, and nutraceutical applications.

## Figures and Tables

**Table 1:** Total phenolics content (TPC) expressed as mg GAE/g of dried plant weight (DW) and total flavonoids content (TFC) expressed as mg RE/g of dried plant weight (DW).

Assay	Solvent	*G. verum*	*F. vulgaris*	*L. salicaria*	*S. montana*	*T. chamaedrys*	*T. montanum*
TPC	aqueous	48.04 ± 0.69 ^c^	88.95 ± 1.33 ^b^	113.56 ± 8.39 ^a^	10.01 ± 0.53 ^e^	85.28 ± 7.37 ^b^	30.66 ± 1.26 ^d^
	ethanol	43.59 ± 1.21 ^c^	88.41 ± 4.11 ^b^	119.09 ± 8.92 ^a^	12.66 ± 0.37 ^e^	77.66 ± 4.35 ^b^	32.64 ± 2.70 ^d^
TFC	aqueous	23.78 ± 0.85 ^d^	65.74 ± 1.56 ^a^	28.46 ± 1.42 ^c^	4.53 ± 0.21 ^f^	36.81 ± 0.80 ^b^	18.06 ± 0.74 ^e^
	ethanol	27.63 ± 0.31 ^c^	66.31 ± 2.66 ^a^	24.96 ± 0.48 ^c^	5.12 ± 0.13 ^e^	58.68 ± 1.55 ^b^	29.59 ± 0.85 ^d^

Values are expressed as mean ± SD (*n* = 3). Different superscript letters within the same assay and solvent indicate statistically significant differences according to Tukey’s HSD test (*p* < 0.05).

**Table 2:** HPLC analysis of polyphenolic compounds in aqueous and ethanol extracts of selected plants in mg/g of dried plant weight (DW).

Assay	Solvent	*G. verum*	*F. vulgaris*	*L. salicaria*	*S. montana*	*T. chamaedrys*	*T. montanum*
Caffeic acid	aqueous	2.34 ± 0.12 ^a^	0.94 ± 0.01 ^b^	n.d.	n.d.	0.84 ± 0.03 ^b^	n.d.
	ethanol	n.d.	1.64 ± 0.03 ^b^	n.d.	2.16 ± 0.04 ^a^	n.d.	n.d.
Gentisic acid	aqueous	n.d.	2.30 ± 0.11 ^a^	n.d.	n.d.	1.77 ± 0.05 ^b^	n.d.
	ethanol	n.d.	n.d.	n.d.	n.d.	n.d.	n.d.
Ferulic acid	aqueous	n.d.	n.d.	n.d.	n.d.	7.33 ± 0.17 ^a^	n.d.
	ethanol	3.09 ± 0.07 ^b^	n.d.	39.12 ± 0.45 ^a^	n.d.	n.d.	0.54 ± 0.02 ^c^
Gallic acid	aqueous	n.d.	6.01 ± 0.18 ^b^	7.95 ± 0.25 ^a^	n.d.	n.d.	1.11 ± 0.02 ^c^
	ethanol	n.d.	1.84 ± 0.01 ^b^	n.d.	n.d.	2.75 ± 0.04 ^a^	n.d.
Sinapic acid	aqueous	n.d.	n.d.	n.d.	n.d.	n.d.	5.78 ± 0.17 ^a^
	ethanol	n.d.	n.d.	n.d.	n.d.	n.d.	10.74 ± 0.17 ^a^
Protocatechuic acid	aqueous	n.d.	n.d.	6.40 ± 0.19 ^a^	n.d.	n.d.	n.d.
	ethanol	n.d.	n.d.	n.d.	n.d.	n.d.	n.d.
Rosmarinic acid	aqueous	n.d.	n.d.	n.d.	n.d.	n.d.	n.d.
	ethanol	n.d.	n.d.	n.d.	n.d.	n.d.	1.66 ± 0.03 ^a^
*p*-Hydroxybenzoic acid	aqueous	0.60 ± 0.01 ^a^	n.d.	n.d.	n.d.	n.d.	n.d.
	ethanol	n.d.	n.d.	n.d.	n.d.	n.d.	n.d.
Syringic acid	aqueous	n.d.	n.d.	n.d.	n.d.	n.d.	n.d.
	ethanol	n.d.	n.d.	n.d.	31.95 ± 0.64 ^a^	2.52 ± 0.03 ^b^	n.d.
Rutin	aqueous	17.28 ± 0.32 ^a^	5.39 ± 0.16 ^b^	n.d.	3.48 ± 0.08 ^c^	n.d.	n.d.
	ethanol	29.25 ± 0.42 ^a^	5.12 ± 0.11 ^b^	n.d.	n.d.	n.d.	n.d.
Myricetin	aqueous	4.65 ± 0.07 ^b^	4.64 ± 0.09 ^b^	7.49 ± 0.21 ^a^	7.36 ± 0.24 ^a^	n.d.	n.d.
	ethanol	7.76 ± 0.18 ^b^	5.69 ± 0.13 ^c^	n.d.	29.57 ± 0.27 ^a^	0.05 ± 0.01 ^d^	n.d.
Luteolin	aqueous	17.57 ± 0.39 ^a^	9.21 ± 0.29 ^b^	n.d.	15.01 ± 0.22 ^a^	n.d.	n.d.
	ethanol	29.14 ± 0.21 ^a^	28.52 ± 0.44 ^a^	n.d.	n.d.	n.d.	n.d.

n.d.—not detected; values are expressed as mean ± SD (*n* = 3). Different superscript letters within the same assay and solvent indicate statistically significant differences according to Tukey’s HSD test (*p* < 0.05).

**Table 3:** Antioxidant activity (expressed as µM TE/g of dried plant weight).

Assay	Solvent	*G. verum*	*F. vulgaris*	*L. salicaria*	*S. montana*	*T. chamaedrys*	*T. montanum*	Standard *
DPPH^●^	aqueous	67.92 ± 2.76 ^e^	328.92 ± 3.00 ^b^	336.81 ± 2.58 ^a^	26.45 ± 0.42 ^f^	319.34 ± 3.79 ^c^	113.15 ± 4.63 ^d^	0.14 ± 0.01
	ethanol	66.45 ± 1.98 ^f^	351.18 ± 12.74 ^b^	378.60 ± 4.89 ^a^	68.34 ± 2.49 ^f^	330.13 ± 9.00 ^c^	123.61 ± 1.24 ^d^	
ABTS^●+^	aqueous	464.64 ± 22.64 ^e^	2300.89 ± 105.25 ^b^	3621.93 ± 77.39 ^a^	133.50 ± 7.88 ^f^	1539.45 ± 55.28 ^c^	565.34 ± 19.49 ^d^	1.06 ± 0.04
	ethanol	291.86 ± 9.99 ^e^	1268.04 ± 20.61 ^b^	2095.97 ± 65.85 ^a^	248.57 ± 3.12 ^f^	1141.50 ± 39.04 ^c^	425.95 ± 1.00 ^d^	
RP	aqueous	67.05 ± 0.18 ^e^	366.27 ± 9.16 ^b^	457.55 ± 11.41 ^a^	16.80 ± 0.26 ^f^	301.54 ± 2.83 ^c^	88.41 ± 0.49 ^d^	1.14 ± 0.03
	ethanol	57.24 ± 2.31 ^e^	493.01 ± 19.58 ^b^	684.06 ± 20.67 ^a^	31.00 ± 0.03 ^f^	321.68 ± 3.56 ^c^	98.83 ± 0.75 ^d^	

DPPH^●^—2,2-diphenyl-1-picrylhydrazyl; ABTS^●+^—2,2-azino-bis-3-ethyl benzathine-zoline-6-sulphonic acid; RP—reducing power, * IC_50_ values of Trolox activity against DPPH and ABTS radicals and reducing power activity; values are expressed as mean ± SD (*n* = 3). Different superscript letters within the same assay and solvent indicate statistically significant differences according to Tukey’s HSD test (*p* < 0.05).

**Table 4:** Antihyperglycemic activity expressed as % inhibition α-glucosidase for final concentration.

Assay	Solvent	*G. verum*	*F. vulgaris*	*L. salicaria*	*S. montana*	*T. chamaedrys*	*T. montanum*
Final concentration mg/mL		3.77	<1	1.87	1.87	1.87	1.87
% inhibition α-glucosidase	aqueous	10.68 ± 0.76 ^e^	100 ^a^	89.27 ± 2.11 ^b^	8.60 ± 0.68 ^f^	29.78 ± 0.90 ^c^	8.61 ± 0.42 ^f^
	ethanol	37.17 ± 1.44 ^c^	100 ^a^	98.82 ± 0.12 ^b^	37.70 ± 1.42 ^c^	9.77 ± 0.92 ^e^	11.04 ± 0.46 ^d^

Values are expressed as mean ± SD (*n* = 3). Different superscript letters within the same assay and solvent indicate statistically significant differences according to Tukey’s HSD test (*p* < 0.05).

**Table 5:** Minimal bactericidal concentration (MBC) and minimal fungicide concentration (MFC) (mg/mL).

Test Organism	Solvent	*G. verum*	*F. vulgaris*	*L. salicaria*	*S. montana*	*T. chamaedrys*	*T. montanum*	Negative Control (Water)	Positive Control (Amoxicillin/Actidione) * (µg/mL)
*E. coli*	aqueous	n.d.	50.00 ± 0.00 ^a^	50.00 ± 0.00 ^a^	50.00 ± 0.00 ^a^	50.00 ± 0.00 ^a^	50.00 ± 0.00 ^a^	n.d.	32.00 ± 0.00
	ethanol	n.d.	50.00 ± 0.00 ^a^	50.00 ± 0.00 ^a^	50.00 ± 0.00 ^a^	50.00 ± 0.00 ^a^	25.00 ± 0.00 ^a^	n.d.	
*S.* Typhimurium	aqueous	n.d.	50.00 ± 0.00 ^a^	25.00 ± 0.00 ^b^	25.00 ± 0.00 ^b^	50.00 ± 0.00 ^a^	25.00 ± 0.00 ^b^	n.d.	32.00 ± 0.00
	ethanol	n.d.	25.00 ± 0.00 ^b^	25.00 ± 0.00 ^b^	25.00 ± 0.00 ^b^	50.00 ± 0.00 ^a^	50.00 ± 0.00 ^a^	n.d.	
*P. aeruginosa*	aqueous	n.d.	50.00 ± 0.00 ^a^	50.00 ± 0.00 ^a^	n.d.	n.d.	n.d.	n.d.	n.d.
	ethanol	n.d.	50.00 ± 0.00 ^a^	25.00 ± 0.00 ^b^	n.d.	n.d.	n.d.	n.d.	
*S. aureus*	aqueous	n.d.	12.50 ± 0.00 ^b^	3.13 ± 0.00 ^c^	50.00 ± 0.00 ^a^	50.00 ± 0.00 ^a^	50.00 ± 0.00 ^a^	n.d.	4.00 ± 0.00
	ethanol	n.d.	12.50 ± 0.00 ^c^	25.00 ± 0.00 ^b^	50.00 ± 0.00 ^a^	50.00 ± 0.00 ^a^	50.00 ± 0.00 ^a^	n.d.	
*E. faecalis*	aqueous	n.d.	25.00 ± 0.00 ^a^	25.00 ± 0.00 ^a^	12.50 ± 0.00 ^b^	n.d.	n.d.	n.d.	8.00 ± 0.00
	ethanol	50.00 ± 0.00 ^a^	25.00 ± 0.00 ^b^	25.00 ± 0.00 ^b^	12.50 ± 0.00 ^c^	n.d.	n.d.	n.d.	
*L. monocytogenes*	aqueous	n.d.	6.25 ± 0.00 ^b^	6.25 ± 0.00 ^b^	25.00 ± 0.00 ^a^	n.d.	n.d.	n.d.	2.00 ± 0.00
	ethanol	n.d.	25.00 ± 0.00 ^a^	3.13 ± 0.00 ^c^	6.25 ± 0.00 ^b^	n.d.	n.d.	n.d.	
*S. cerevisiae*	aqueous	n.d.	n.d.	n.d.	n.d.	n.d.	n.d.	n.d.	0.5 ± 0.00
	ethanol	n.d.	n.d.	n.d.	n.d.	n.d.	n.d.	n.d.	
*C. albicans*	aqueous	n.d.	n.d.	n.d.	n.d.	n.d.	n.d.	n.d.	2.00 ± 0.00
	ethanol	n.d.	n.d.	n.d.	n.d.	n.d.	n.d.	n.d.	
*A. brasiliensis*	aqueous	n.d.	n.d.	n.d.	n.d.	n.d.	n.d.	n.d.	4.00 ± 0.00
	ethanol	n.d.	n.d.	n.d.	n.d.	n.d.	n.d.	n.d.	

n.d.—not detected; * amoxicillin was a positive control for bacteria, while actidione was a positive control for fungi and yeasts; values are expressed as mean ± SD (*n* = 3). Different superscript letters within the same assay and solvent indicate statistically significant differences according to Tukey’s HSD test (*p* < 0.05).

**Table 6:** Literature review compared with results of this study (TS) of total phenolic (TPC) and total flavonoid (TFC) content in selected plants.

Sample	Solvent	TPC	TFC	Reference
*G. verum*	Methanol	2.44–5.16 mg GAE/g DE	6.38–17.96 μg QE/g DE	[21]
	Ethanol	2.6 g GAE/100 g DW	5.21 g RE/100 g DW	[22]
	Methanol	118.13 mg GAE/g DE	9.05 mg RE/g DE	[23]
	Methanol	86.40 mg GAE/g DE	23.11 mg QE/g DE	[24]
	Acetone	27.2 mg GAE/g extract	7.3 mg QE/g extract	[25]
	Ethanol	20.7 mg GAE/g extract	5.6 mg QE/g extract	[25]
	Ethanol	1.30 mg GAE/g DE	1.42 mg CE/g DE	[26]
	Ethyl acetate	1.39 mg GAE/g DE	1.37 mg CE/g DE	[26]
	Aqueous	48.04 mg GAE/g of DW	23.78 mg RE/g of DW	TS
	Ethanol	43.59 mg GAE/g of DW	27.63 mg RE/g of DW	TS
*F. vulgaris*	Acetone	35.52 mg GAE/g DW	-	[27]
	Methanol	49.91 mg GAE/g DW	-	[27]
	Water	16.23 mg GAE/g DW	-	[27]
	Ethanol	1163 mg GAE/L extract	-	[28]
	Aqueous	88.95 mg GAE/g of DW	65.74 mg RE/g of DW	TS
	Ethanol	88.41 mg GAE/g of DW	66.31 mg RE/g of DW	TS
*L. salicaria*	Methanol	201.50 mg GAE/g extract	43.36 mg QE/g extract	[29]
	Aqueous	113.56 mg GAE/g of DW	28.46 mg RE/g of DW	TS
	Ethanol	119.09 mg GAE/g of DW	24.96 mg RE/g of DW	TS
*S. montana*	Methanol	51 mg GAE/g DW	16.3 mg QE/g DW	[30]
	Aqueous	10.01 mg GAE/g of DW	4.53 mg RE/g of DW	TS
	Ethanol	12.66 mg GAE/g of DW	5.12 mg RE/g of DW	TS
*T. chamaedrys*	50% EtOH by MAE	67.35 mg GAE/g extract	53.01 mg RE/g extract	[31]
	Aqueous	85.28 mg GAE/g of DW	36.81 mg RE/g of DW	TS
	Ethanol	77.66 mg GAE/g of DW	58.68 mg RE/g of DW	TS
*T. montanum*	50% EtOH by MAE	73.60 mg GAE/g extract	65.31 mg RE/g extract	[31]
	Aqueous	30.66 mg GAE/g of DW	18.06 mg RE/g of DW	TS
	Ethanol	32.64 mg GAE/g of DW	29.59 mg RE/g of DW	TS

GAE—Gallic acid equivalent; QE—Quercetin equivalent; CE—catechin equivalent; RE—rutin equivalent; DW—dry weight; DE—dry extract; TS—this study.

**Table 7:** Literature review compared with results of this study (TS) of the polyphenolic profiles of selected plants.

Sample	Extract	HPLC Analysis	Reference
*G. verum*	Ethanol (mg/100 g DW)	*p*-coumaric acid (0.983), isoquercitrin (78.021), rutin (804.262), quercitrin (4.291), quercetin (2.651), kaempferol (3.069)	[22]
	Ethanol (%)	chlorogenic acid (32.60), coumaric acid (2.02), ferulic acid (0.65), rutin (12.14), isoquercitrin (3.93), quercetin (0.13)	[42]
	Ethanol (μg/mL)	chlorogenic acid (8.027), 4-*O*-caffeoylquinic acid (0.172), isoquercitrin (17.765), rutin (14.811), quercetol (1.275), luteolin (0.260)	[26]
	Ethyl acetate (μg/mL)	chlorogenic acid (10.216), 4-*O*-caffeoylquinic acid (0.096), isoquercitrin (20.384), rutin (1.896), rutin (1.896), quercitrin (6.722), quercetol (0.779), luteolin (0.191)	[26]
	Water (μg/mL)	chlorogenic acid (2.063), rutin (0.560)	[43]
	Aqueous (mg/g of DW)	caffeic acid (2.34), *p*-hydroxybenzoic acid (0.60), rutin (17.28), myricetin (4.65), luteolin (17.57)	TS
	Ethanol (mg/g of DW)	ferulic acid (3.09), rutin (29.25), myricetin (7.76), luteolin (29.14)	TS
*F. vulgaris*	Methanol (mg/100 g DW)	gallic acid (58), catechin (208.9), ellagic acid (28.6), hyperoside (227.6), luteolin-7-glucoside (145.3), spiraeoside (304.8), astragalin (83.0)	[27]
	Methanol (µg/g extract)	gallic acid (925.67), ellagic acid (525.37), catechin (202.36), epicatechin (181.61), quercetin (118.32), fumaric acid (65.78), chlorogenic acid (47.18), *p*-coumaric acid (11.39), rosmarinic acid (6.68), ferulic acid (0.28)	[34]
	Acetone (mg/100 g DW)	catechin (10.8), ellagic acid (134.4), hyperoside (69.7), spiraeoside (267.1), astragalin (17.7)	[27]
	Water (mg/100 g DW)	gallic acid (14.5), catechin (18.3), ellagic acid (198.5), spiraeoside (134.2)	[27]
	Ethanol (µg/mL extract)	gallic acid (20.97), phloroglucinic acid (1.29), hydroxybenzoic acid (0.71), catechin (1.40), resorcylic acids (0.57), hydroxybenzaldehyde (0.29), caffeic acid (0.49), hydroxycinnamic acid (0.69), ellagic acid (2.74), rosmarinic acid (0.14), astragalin (11.26), quercetin (37.72), naringenin (0.45), luteolin (0.95), kaempferol (10.57)	[28]
	Methanol (mg/100 g DW)	hyperoside (516.7), astragalin (393.8), spireaoside (1113.6), kaempferol (231.3), quercetin (83.2), epigallocatechin (49.7), catechin (70.9), ellagic (210.0), gallic (477.9), syryngic (344.7), salicylic (46.6)	[44]
	Water (mg/g)	spiraeoside (55.67), hyperoside (5.12), hyperoside 2″-*O*-gallate (3.8), isoquercitrin (13.24), isoquercitrin 2″-*O*-gallate (6.3), astragalin (11.22), astragalin 2″-*O*-gallate (4.58), kaempferol 4′-*O*-β-D-glucoside (4.91), gallic acid (10.22), ellagic acid (3.8), salicylic acid (3.5), tellimagrandin II (15.80)	[45]
	Aqueous (mg/g of DW)	caffeic acid (0.94), gentisic acid (2.30), gallic acid (6.01), rutin (5.39), myricetin (4.64), luteolin (9.21)	TS
	Ethanol (mg/g of DW)	caffeic acid (1.64), gallic acid (1.84), rutin (5.12), myricetin (5.69), luteolin (28.52)	TS
*L. salicaria*	Methanol (mg/g of DE)	gallic acid (2.1), ellagic acid (2.6), orientin (1.68), isoorientin (1.84), vitexin (3.93)	[29]
	Aqueous (mg/g of DW)	gallic acid (7.95), protocatechuic acid (6.40), myricetin (7.49)	TS
	Ethanol (mg/g of DW)	ferulic acid (39.12)	TS
*S. montana*	Ethanol (mol/g DP)	caffeic acid (3.13), luteolin-7-*O*-glucoside (17.44), kaempferol-3-*O*-rutinoside (1.32), ferulic acid (0.83), kaempferol-3-*O*-glucoside (2.97), rosmarinic acid (20.34)	[46]
	Aqueous (mg/g of DW)	rutin (3.48), myricetin (7.36), luteolin (15.01)	TS
	Ethanol (mg/g of DW)	caffeic acid (2.16), syringic acid (31.95), myricetin (29.57)	TS
*T. chamaedrys*	Methanol (% of DW)	caffeic acid (0.02), 5-caffeoylquinic acid (0.1), forsythoside b (39.0), verbascoside (4.5), teucreoside (2.0), allysonoside (1.5), poliumoside (0.2), luteolin 7-*O*-rutinoside (1.6), luteolin 7-*O*-glucoside (0.3), quercetin 3-*O*-rutinoside (0.4), apigenin 7-*O*-rutinoside (0.5), apigenin 7-*O*-glucoside (0.6), diosmetin 7-*O*-rutinoside (0.3), apigenin (0.03), cirsiliol (0.6), cirsimaritin (0.04)	[41]
	Aqueous (mg/g of DW)	caffeic acid (0.84), gentisic acid (1.77), ferulic acid (7.33)	TS
	Ethanol (mg/g of DW)	gallic acid (2.75), syringic acid (2.52), myricetin (0.05)	TS
*T. montanum*	Methanol (% of DW)	caffeic acid (0.04), 5-caffeoylquinic acid (0.2), caerulescenoside (7.0), castanoside a (1.1), echinacoside (2.4), forsythoside B (10.2), verbascoside (2.0), samioside (1.7), luteolin 7-*O*-rutinoside (0.6), luteolin 7-*O*-glucoside (0.1), quercetin 3-*O*-rutinoside (1.0), diosmetin 7-*O*-rutinoside (0.4), luteolin (0.1), apigenin (0.04), cirsiliol (0.7), cirsimaritin (0.5)	[41]
	Aqueous (mg/g of DW)	gallic acid (1.11), sinapic acid (5.78)	TS
	Ethanol (mg/g of DW)	ferulic acid (0.54), sinapic acid (10.74), rosmarinic acid (1.66)	TS

GAE—Gallic acid equivalent; QE—Quercetin equivalent; CE—catechin equivalent; RE—rutin equivalent; DW—dry weight; DE—dry extract.

**Table 8:** Literature review compared with results of this study (TS) of antioxidant activity of selected plants.

Sample	Antioxidant Activity	Extract	Reference
*G. verum*	DPPH: 3.10–8.04 μg/mL (IC_50_)	Methanol	[21]
	DPPH: 105.43 μg/mL (IC_50_)	Ethanol	[22]
	DPPH: 26.97 µg/mL of extract (IC_50_) ABTS: 125.14 mg Trolox/g dry extract RP: 70.31 μg of AA/mL of extract	Methanol	[23]
	DPPH: 0.136 mg/mL (EC_50_)	Ethanol	[26]
	DPPH: 0.074 mg/mL (EC_50_)	Ethyl acetate	[26]
	DPPH: 4.6 mg QE/g plant TEAC: 0.082 mg QE/g plant	Ethanol	[42]
	DPPH: 67.92 µM TE/g ABTS: 464.64 µM TE/g RP: 67.05 µM TE/g	Aqueous	TS
	DPPH: 66.45 µM TE/g ABTS: 291.86 µM TE/g RP: 57.24 µM TE/g	Ethanol	TS
*F. vulgaris*	DPPH: 6 mmol Trolox/L of extract	Ethanol	[28]
	DPPH: 9.10 μg/mL (IC_50_) FRAP: 7.72 (mmol Fe^2+^/g)	Water	[45]
	DPPH: 328.92 µM TE/g ABTS: 2300.89 µM TE/g RP: 366.27 µM TE/g	Aqueous	TS
	DPPH: 351.18 µM TE/g ABTS: 1268.04 µM TE/g RP: 493.01 µM TE/g	Ethanol	TS
*L. salicaria*	DPPH: 15.78 μg/mL (IC_50_) ABTS: 23.49 μg/mL (IC_50_) RP: 438.37 mg Trolox/g extract	Methanol	[29]
	DPPH: 336.81 µM TE/g ABTS: 3621.93 µM TE/g RP: 457.55 µM TE/g	Aqueous	TS
	DPPH: 378.60 µM TE/g ABTS: 2095.97 µM TE/g RP: 684.06 µM TE/g	Ethanol	TS
*S. montana*	FRAP: 93 mg QE/g	Methanol	[30]
	DPPH: 26.45 µM TE/g ABTS: 133.50 µM TE/g RP: 16.80 µM TE/g	Aqueous	TS
	DPPH: 68.34 µM TE/g ABTS: 248.57 µM TE/g RP: 31.00 µM TE/g	Ethanol	TS
*T. chamaedrys*	ABTS: 73.32 mg TE/g extract	Ethanol by MAE	[31]
	DPPH: 319.34 µM TE/g ABTS: 1539.45 µM TE/g RP: 301.54 µM TE/g	Aqueous	TS
	DPPH: 330.13 µM TE/g ABTS: 1141.50 µM TE/g RP: 321.68 µM TE/g	Ethanol	TS
*T. montanum*	ABTS: 74.29 mg TE/g extract	Ethanol by MAE	[31]
	DPPH: 113.15 µM TE/g ABTS: 565.34 µM TE/g RP: 88.41 µM TE/g	Aqueous	TS
	DPPH: 123.61 µM TE/g ABTS: 425.95 µM TE/g RP: 98.83 µM TE/g	Ethanol	TS

QE—quercetin equivalents; AA—ascorbic acid.

**Table 9:** Literature review compared with results of this study (TS) of antihyperglycemic activity of selected plants.

Sample	α-Glucosidase Inhibition	Extract	Reference
*G. verum*	+	Ethanol	[52]
	10.68%	Aqueous	TS
	37.17	Ethanol	TS
*F. vulgaris*	100%	Aqueous	TS
	100%	Ethanol	TS
*L. salicaria*	+	Ethyl ether	[54]
	89.27%	Aqueous	TS
	98.82%	Ethanol	TS
*S. montana*	8.60%	Aqueous	TS
	37.70%	Ethanol	TS
*T. chamaedrys*	3.84 mmol ACAE/g extract	50% EtOH by MAE	[31]
	29.78%	Aqueous	TS
	9.77%	Ethanol	TS
*T. montanum*	4.55 mmol ACAE/g extract	50% EtOH by MAE	[31]
	10.97%	Aqueous	[60]
	31.52%	Acetone	[60]
	32.54%	Ethanol	[60]
	8.61%	Aqueous	TS
	11.04%	Ethanol	TS

ACAE—acarbose equivalents; +—indicates the presence of α-glucosidase inhibitory activity of apigenin 7-*O*-(2,4-di-*O*-acetyl)-α-L-rhamnopyranosyl-(1→6)-β-D-glucopyranoside; the IC_50_ value refers only to the compound isolated from *G. verum*; TS—this study

**Table 10:** Literature review compared with results of this study (TS) of antimicrobial activity of selected plants.

Sample	Tested Microorganisms	Extract	Reference
*G. verum*	MIC (mg/mL): *Streptococcus pyogenes* (30), *S. aureus* (30), *E. coli* (30), *P. aeruginosa* (no activity)	Water	[43]
	MBC (mg/mL): *E. coli* (50)	Aqueous	TS
	MBC (mg/mL): *E. coli* (50), *E. faecalis* (50)	Ethanol	TS
*F. vulgaris*	-	-	-
	MBC (mg/mL): *E. coli* (50), *S.* Typhimurium (50), *P. aeruginosa* (50), *S. aureus* (12.5), *E. faecalis* (25), *L. monocytogenes* (6.25)	Aqueous	TS
	MBC (mg/mL): *E. coli* (50), *S.* Typhimurium (25), *P. aeruginosa* (50), *S. aureus* (12.5), *E. faecalis* (25), *L. monocytogenes* (25)	Ethanol	TS
*L. salicaria*	MIC (mg/mL): *Micrococcus lysodeikticus* (1.25), *Ent. faecalis* (5), *E. coli* (2.5), *K. pneumoniae* (2.5), *P. aeruginosa* (5), *Bacillus cereus* (2.5), *B. subtilis* (0.625), *St. epidermidis* (5), *S. aureus* (0.625), *S. enteritidis* (1.25), *S.* Typhimurium (2.5), *C. albicans* (5), *Fusarium oxysporum* (10), *A. brasiliensis* (>20), *Alternaria alternata* (>20), *Doratomyces stemonitis* (>20), *Trichoderma. longibrachiatum* (20), *T. harzanum* (20), *Penicillium canescens* (20), *Pe. cyclopium* (20)	Methanol	[29]
	MIC (mg/mL): *S. aureus* (0.5), *Proteus mirabilis* (1.00), *M. luteus* (1.00)	Methanol	[65]
	MBC (mg/mL): *E. coli* (50), *S.* Typhimurium (25), *P. aeruginosa* (50), *S. aureus* (3.13), *E. faecalis* (25), *L. monocytogenes* (6.25)	Aqueous	TS
	MBC (mg/mL): *E. coli* (50), *S.* Typhimurium (25), *P. aeruginosa* (25), *S. aureus* (25), *E. faecalis* (25), *L. monocytogenes* (3.13)	Ethanol	TS
*S. montana*	MIC (mg/mL): *E. coli* (60), *P. aeruginosa* (30), *S. enteritidis* (30), *S. aureus* (60)	Ethanol	[66]
	MIC (mg/mL): *E. coli* (20), *S. aureus* (10), *Ent. faecalis* (10), *P. aeruginosa* (5), *B. subtilis* (1.25), *B. cereus* (0.625) *Sarcina lutea* (1.25), *S. enterica* (20), *Pr. mirabilis* (5)	Acetone	[63]
	MIC (mg/mL): *E. coli* (20), *S. aureus* (10), *Ent. faecalis* (20), *P. aeruginosa* (2.5), *B. subtilis* (1.25), *B. cereus* (0.625) *S. lutea* (1.25), *S. enterica* (20), *Pr. mirabilis* (5)	Ethyl acetate	[63]
	MIC (mg/mL): *E. coli* (10), *S. aureus* (20), *Ent. faecalis* (10), *P. aeruginosa* (10), *B. subtilis* (1.25), *B. cereus* (0.625) *S. lutea* (2.5), *S. enterica* (20), *Pr. mirabilis* (1.25)	Methanol	[63]
	MBC (mg/mL): *E. coli* (50), *S.* Typhimurium (25), *S. aureus* (50), *E. faecalis* (12.50), *L. monocytogenes* (25)	Aqueous	TS
	MBC (mg/mL): *E. coli* (50), *S.* Typhimurium (25), *S. aureus* (50), *E. faecalis* (12.50), *L. monocytogenes* (6.25)	Ethanol	TS
*T. chamaedrys*	MIC (mg/mL): *B. subtilis* (300), *Pe. fluorescens* (150), *Xantomonas campestris* (150), *Erwinia amylovora* (300), *Er. carotovora* (300), *C. utilis* (300)	Ethanol	[62]
	MIC (mg/mL): *E. coli* (5), *S. aureus* (0.078), *P. aeruginosa* (1.25), *C. albicans* (>20), *A. niger* (>20)	Methnol	[61]
	MIC (mg/mL): *E. coli* (20), *S. aureus* (5), *P. aeruginosa* (20), *C. albicans* (>20), *A. niger* (10)	Acetone	[61]
	MIC (mg/mL): *E. coli* (10), *S. aureus* (0.6), *P. aeruginosa* (10), *C. albicans* (>10), *A. niger* (10)	Ethyl acetate	[61]
	MBC (mg/mL): *E. coli* (50), *S.* Typhimurium (50), *S. aureus* (50)	Aqueous	TS
	MBC (mg/mL): *E. coli* (50), *S.* Typhimurium (50), *S. aureus* (50)	Ethanol	TS
*T. montanum*	MIC (mg/mL): *P. aeruginosa* (>2.5), *E. coli* (>10), *S. aureus* (1.0), *S. lutea* (10), *Bacillus* sp. (7.5)	Ethyl acetate; n-butanol	[67]
	MIC (mg/mL): *P. aeruginosa* (10), *E. coli* (10), *S. aureus* (5), *S. lutea* (10), *Bacillus* sp. (10)	Chloroform	[67]
	MIC (mg/mL): *P. aeruginosa* (>10), *E. coli* (>10), *S. aureus* (>10), *S. lutea* (10), *Bacillus* sp. (10)	Petroleum ether	[67]
	MIC (mg/mL): *E. coli* (5), *S. aureus* (0.15), *P. aeruginosa* (2.5), *C. albicans* (>20), *A. niger* (>20)	Methnol	[61]
	MIC (mg/mL): *E. coli* (10), *S. aureus* (0.15), *P. aeruginosa* (5), *C. albicans* (>20), *A. niger* (20)	Acetone	[61]
	MIC (mg/mL): *E. coli* (>10), *S. aureus* (0.3), *P. aeruginosa* (10), *C. albicans* (>10), *A. niger* (>10)	Ethyl acetate	[61]
	MBC (mg/mL): *E. coli* (50), *S.* Typhimurium (25), *S. aureus* (50)	Aqueous	TS
	MBC (mg/mL): *E. coli* (25), *S.* Typhimurium (50), *S. aureus* (50)	Ethanol	TS

MIC—Minimal inhibitory concentration, MBC—minimal bactericidal concentration; TS—this study.

## Data Availability

The original contributions presented in this study are included in the article. Further inquiries can be directed to the corresponding author.

## Abbreviations

The following abbreviations are used in this manuscript:

AA	Ascorbic Acid
ACAE	Acarbose Equivalents
CE	Catechin Equivalent
DE	Dry Extract
DW	Dry Weight
GAE	Gallic acid equivalent
QE	Quercetin equivalent
RE	Rutin Equivalent
TFC	Total Flavonoid Compounds
TPC	Total Phenolic Compounds
TS	This Study
